# Promoting Collaborative Goal Setting for Cancer Prevention Among Primary Care Patients Through mHealth: Mixed Methods Evaluation of a New App

**DOI:** 10.2196/22510

**Published:** 2021-07-14

**Authors:** Daniel Resnick, Marilyn M Schapira, Jazmine M Smith, Allison Bautista, Chang Xu, Liz Jones, Jaya Aysola

**Affiliations:** 1 Perelman School of Medicine, University of Pennsylvania Philadelphia, PA United States; 2 Penn Medicine Center for Health Equity Advancement Office of the Chief Medical Officer University of Pennsylvania Health System Philadelphia, PA United States; 3 Leonard Davis Institute of Health Economics University of Pennsylvania Philadelphia, PA United States; 4 Division of General Internal Medicine Department of Medicine Perelman School of Medicine, University of Pennsylvania Philadelphia, PA United States; 5 The Center for Health Equity Research and Promotion Michael J Crescenz VA Medical Center Philadelphia, PA United States; 6 Office of Inclusion and Diversity Perelman School of Medicine, University of Pennsylvania Philadelphia, PA United States; 7 Transmogrify Conshohocken, PA United States

**Keywords:** mHealth, cancer prevention, goal setting, social networks, health disparities, mobile phone

## Abstract

**Background:**

Many newly diagnosed cancers are associated with modifiable lifestyle behaviors, such as diet, exercise, smoking cessation, and maintaining a healthy weight. However, primary care providers rarely discuss cancer prevention behaviors with their patients.

**Objective:**

This study aims to assess the usability, acceptability, and user engagement of the Healthier Together mobile app, which is designed to promote cancer prevention behaviors among non-Hispanic Black primary care patients, by using social networks and goal-setting theories of behavior change.

**Methods:**

In an 8-week pilot study, we enrolled primary care patients (N=41) and provided them with a cancer prevention mobile app that allowed them to select, track, and share progress on cancer prevention goals with other users. App usability was assessed using the System Usability Scale. We assessed the app’s acceptability by qualitatively analyzing open-ended responses regarding participants’ overall experience with the app. We assessed participants’ engagement by analyzing the built-in data capture device, which included the number of times participants checked in (out of a maximum of 8) during the study.

**Results:**

The mean age of the 41 participants was 51 years (SD 12), and 76% (31/41) were women. App use data were captured from all participants, and 83% (34/41) completed the exit survey and interview. The mean System Usability Scale score was 87 (SD 12; median 90; IQR 78-95). The analysis of open-ended responses revealed several key themes, and participants complemented the app’s ease of use and health behavior–promoting features while also commenting on the need for more feedback and social interactions through the app. On average, participants checked in 5.7 times (SD 2.7) out of 8 possible opportunities. Of the 41 participants, 76% (31/41) checked in during at least 4 of the 8 weeks. Secondary analyses revealed that participants often accomplished their set goals (mean 5.1, SD 2.7) for each week. The qualitative analysis of comments given by participants within the app after each weekly check-in revealed several themes on how the app assisted participants in behavioral change, highlighting that some participants created exercise programs, ate healthier foods, lost a significant amount of weight, and stopped smoking during this study.

**Conclusions:**

The implementation of a mobile cancer prevention goal–setting app in a primary care setting was feasible, and the app achieved high usability, acceptability, and engagement among participants. User feedback revealed an influence on health behaviors. These findings suggest the promise of the Healthier Together app in facilitating behavioral change to reduce cancer risk among non-Hispanic Black primary care patients.

## Introduction

### Background

Each year, more than 1.7 million Americans are diagnosed with cancer [1]. A diet rich in fruits and vegetables [2,3], physical activity [4], smoking cessation [5], and maintaining a healthy body weight reduce the relative risk of developing numerous cancers. For instance, obesity alone is now thought to be associated with nearly 50% of newly diagnosed cancers in the United States in those aged 65 years or younger [6]. Despite these staggering statistics, many primary care patients do not adopt cancer prevention behaviors, and primary care providers (PCPs) rarely discuss these behaviors with their patients. Patients wish to discuss cancer prevention with their PCPs [7]; however, PCPs often cite competing priorities, limited time, and the lack of resources as reasons for not engaging patients in cancer prevention discussions [8].

Although PCPs do not routinely engage their patients in promoting healthy behaviors, mobile phone apps have emerged as a tool for promoting healthy behaviors outside of the clinical realm [9,10]. However, current behavior change apps available for public use rarely provide a theoretical explanation for how they motivate behavior or evidence to support their ability to change behaviors [11,12]. For example, many apps invite users to set behavior goals; however, few ask users to select specific time-bound goals with periodic reviews, although these latter features increase the chances of goal attainment [11,13]. Moreover, a number of apps provide “unspecified social support” [12], without encouraging users to provide more practical and emotional support to one another, despite evidence suggesting that social reinforcements increase the adoption of health behaviors [14]. A final limitation of mobile apps is that there are few apps designed specifically for minority populations, even with evidence that minority patients use their mobile phones to engage with a broad range of health materials more frequently than White patients [15,16]. Despite this, minority populations remain underrepresented in studies involving health and technology [17].

### Objectives

The objective of this study is to evaluate the beta version of an evidence-based mobile app—Healthier Together—which is designed to address the limitations mentioned above and promote cancer prevention behaviors in predominantly minority populations recruited in a primary care setting. Our primary aim is to assess the usability, acceptability, and user engagement of the app during a 2-month study. Our secondary aim is to assess the relationships between app engagement, participant baseline characteristics, and participant health behavior.

## Methods

### Previous Work on Healthier Together

#### Key Features and Theoretical Basis

Healthier Together leverages both goal-setting and social network theories to motivate behavior change through 3 key features, as described later. These features aim to enhance, rather than replace, the role of a PCP in promoting cancer prevention behaviors. These features provide a starting point for patients to learn about the connection between behaviors, such as diet and exercise, and cancer risk, to set and track cancer prevention goals, and to share their progress with other app users. Patients using this app may build upon these actions and initiate cancer prevention discussions with their PCPs that would otherwise not have occurred.

The first feature asks app users to select a predetermined cancer prevention SMART (specific, measurable, achievable, realistic, and time-bound) goal [18] within 1 of the 4 goal categories: diet, activity, weight tracking, and smoking cessation. The app presents goals adapted from the recommendations of the American Cancer Society on diet, exercise, smoking cessation, and maintaining a healthy weight [19,20], linking directly to the American Cancer Society resources. The app provides the option to customize a person’s goal upon selection and modify any selected goal weekly. This key feature leverages existing evidence, demonstrating that goal setting motivates behavioral change through directing intention, building self-efficacy, fostering motivation, and serving as a reference point to invoke loss aversion [13,21,22]. Moreover, a meta-analysis of previous cancer prevention studies found that interventions that incorporated goal-setting strategies were significantly more effective in reducing dietary fat consumption and increasing fruit and vegetable consumption compared with those that did not, with small to moderate differences in effect size [23-25].

To encourage accountability to their selected goal, app users receive a weekly check-in text inviting them to mark whether they succeeded or failed to complete the goal for the week. While checking in, they can also leave a comment reflecting on their progress and change their goals if appropriate. App users can visualize their goal progress on an individual profile page and on a progress board that is potentially visible to other users. Evidence supports the use of goal reminders, tracking, and reflections as important behavior change techniques that may slowly encourage users to develop healthy habits [26].

The third key feature allows Healthier Together app users to communicate and share goal progress with one another. App users can share this information with all app users or with users they invite directly into the app. There is also an option for users to keep all information private. If app users select to share their goal information, they can see other users’ successes on a progress board and send encouraging messages to one another. The purpose of this feature is to allow users to receive social rewards and feedback. It draws upon research that demonstrates social networks with social reinforcements from multiple social ties, as compared with single ties, are associated with greater adoption of health behaviors and health-related knowledge [14,27].

**Figure 1:**
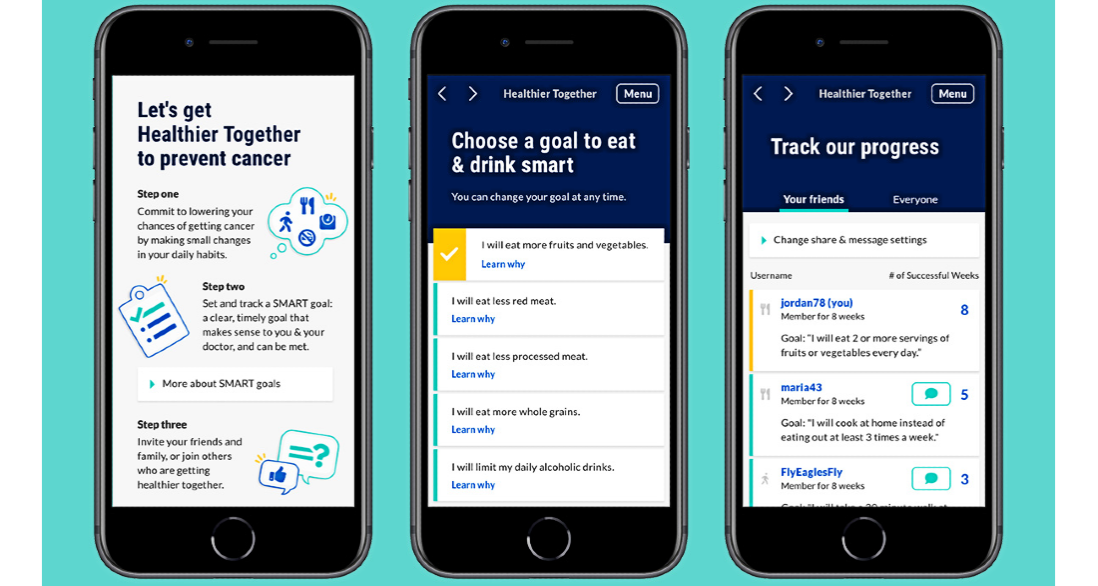
Screenshots of the Healthier Together app.

The Healthier Together app (screenshot is given in Figure 1) was developed primarily for minority populations, as cancer disproportionately impacts racial/ethnic minorities in the United States [28], with additional disparities in behaviors related to cancer prevention, such as smoking [29], obesity [30], diet [31], and exercise [31]. Minority patients use mobile phones for health-related content more than their White counterparts [15,16], suggesting a mobile app may be one strategy to reduce the aforementioned disparities. In addition, prior work suggests that cancer prevention strategies involving some form of social support are more effective in changing behaviors in racial/ethnic minorities than non-Hispanic White individuals [32]. There is also evidence that minorities have denser social networks, with more reliable and frequent activation of informal social support [33,34].

#### Prior Testing and App Development Team

In keeping with best practices for health app development [35], we previously conducted iterative testing [36] with 33 non-Hispanic Black primary care patients to develop this beta version of Healthier Together. Consistent with Healthier Together’s theoretical basis of enabling goal setting and developing social networks, testing revealed that end users valued app features that assist with tracking and sharing progress on health goals. These end users understood that the behaviors promoted by Healthier Together may help prevent other noncommunicable diseases, but valued the specific connection between lifestyle behaviors and cancer prevention and even asked to emphasize the connection further. Moreover, prior evidence reveals that framing the rationale for adopting healthy behaviors as a long-term gain to reduce cancer risk is effective [37-39]. The app itself was developed by Transmogrify, a firm that helps create, build, and grow digital products. The diverse research team (including DR, JMS, AB, MMS, and JA), which has expertise in qualitative methods, communicated closely with LJ, who works for Transmogrify and helped conduct user testing.

### Study Overview

This 8-week mixed method intervention involved 3 key components: (1) a baseline visit that included an in-person structured interview followed by installation of the mobile app on the participant’s phone and instructions on how to select a goal, choose share settings, and invite other social ties; (2) weekly text messages reminding participants to check in, share goal progress, and invite friends and family members; and (3) an exit telephone-structured interview at the end of 8 weeks.

#### Study Population and Recruitment

We recruited patients from 2 internal medicine primary care clinics in Philadelphia, a nonprobabilistic purposive sample of non-Hispanic Black patients that met our strict eligibility criteria detailed below. From September 2019 through October 2019, authors DR, JMS, and AB identified potential participants in clinics’ waiting rooms and invited these individuals to formally screen for the study in a private room after their appointments. Once in the private room, this research team screened potential participants to confirm eligibility, informed them of the study’s app testing goals, and obtained consent to participate in the study.

To be eligible for the study, participants had to be aged more than 18 years, self-identify as non-Hispanic Black, speak English, own a smartphone, be a patient of one of the two clinics, be able to provide informed consent, and should not have participated in previous rounds of app testing. We targeted a sample size of 40 participants to obtain thematic saturation while soliciting feedback during the exit interviews [40].

Participants were incentivized US $40 to complete the baseline, in-person, 40-minute enrollment process and interview and US $60 for completing the exit 45-minute, telephone interview in an effort to maximize recruitment and minimize attrition. Authors JMS and AB attempted to contact each participant up to three times for the exit interview to further minimize loss to follow-up. There were no incentives for app use. The University of Pennsylvania Institutional Review Board approved the protocol for this study.

#### Study Procedure and Data Collection

After consenting to the study, we conducted a 40-minute baseline structured interview to collect participants’ baseline characteristics, as detailed later. The interview included up to 79 close-ended, validated, and previously operationalized survey questions and up to 40 open-ended questions. The interview team inputted all close- and open-ended responses into REDCap (Research Electronic Data Capture; REDCap Consortium) verbatim [41].

The research team then helped the participants download the app, demonstrated each of the features detailed above, and assisted participants with selecting their initial health goal, determining their goal share settings, and sending invitations to their family and friends to download the app.

After downloading the app, participants received a weekly text message inviting them to log on to the app and check in to report whether they accomplished their goal. Participants could also log on to the app outside of the weekly check-in to explore their profile page, track other user progress (if they shared their progress with others), and read information about the relationship between lifestyle behaviors and cancer. In addition, participants received text messages to remind them to share the app with other friends and family. Participants’ check-ins and other activities in the app were recorded through a built-in data capture device and served as our chief measures of app engagement, as described later.

After 8 weeks of app use, DR, JMS, and AB contacted the participants to complete a 45-minute, telephonic exit interview, primarily assessing the participants’ opinions on app usability and acceptability. The exit interview also repeated questions from the baseline interview regarding cancer prevention knowledge and behaviors.

### Data Measurement and Analysis

#### Baseline Characteristics

The baseline interview assessed participants’ demographics, namely, age and sex, and close-ended questions on the following topics: technology use, as defined by what phone participants use and how often they use it; participants’ comfort in sharing health information, as defined by whether participants have shared health information over the web, shared health information with social ties, or shared health goals with social ties; and participants’ current health habits, as defined by whether participants currently have health goals or use some methods to track their health.

#### Primary Outcome Measures

##### Usability

We assessed app usability by asking participants during the exit interview to respond to the validated System Usability Scale (SUS), which provides a usability score of 0 to 100 for various technology products [42]. A score >70 is generally considered above average. We also assessed specific feature usability characteristics, namely, the feature’s ease of use and ability to impart new information, with a modified subset of 5-point Likert scale questions from the SUS.

##### Acceptability

We assessed app acceptability during the structured exit interview with up to 27 open-ended questions about the participants’ overall experience with the app. These questions allowed users to expand upon their close-ended responses and offer more information about what appealed (or did not appeal) to them in the app.

##### Engagement

We assessed engagement with the app in weekly intervals using built-in data capture device. The following measures were captured: (1) the number of check-ins to report goal progress, with the maximum allowable number of check-ins for each participant being 8 during the study; (2) goal type selected; (3) share settings selected; (4) messages to other users; (5) comments on their progress; and (6) any modifications to goals and share setting during the study period.

#### Secondary Outcome Measures

We examined the following measures to assess the implications of engagement with the app: (1) goal success count, defined as the total number of weeks a participant reported accomplishing his or her goal, with the maximum being 8, as recorded by the app’s data capture; (2) goal reflections from participants’ open-ended comments explaining the significance of goal success or failure during each check-in, as recorded by the app’s data capture; (3) change in cancer prevention knowledge using survey questions that asked how important diet, exercise, smoking, and maintaining a healthy weight are to one’s cancer risk using a 5-point Likert scale for each behavior, which were assessed at the baseline and exit interviews; and (4) change in participants’ self-reported cancer prevention behaviors, specifically self-reported diet, exercise, smoking status, and alcohol using validated questions asked at the baseline and exit interviews [43,44].

#### Analysis

First, we examined participants’ baseline characteristics by tabulating the distributions or frequencies of participants’ demographics, current technology use, comfort sharing health information with social ties, current health goals, and methods of health tracking. Second, we characterized app usability, app acceptability, and the nature and frequency of app engagement. Finally, in secondary analyses, we examined associations between app engagement and participant characteristics and goal success and changes in self-reported cancer prevention knowledge and behaviors.

We assessed app usability quantitatively by calculating each participant’s SUS score based on validated criteria [45] and then determining the distributions of SUS scores for all participants who completed an exit survey. We also calculated the distribution of Likert scale responses, ranging from strongly disagree to strongly agree, to statements assessing specific app features.

We assessed app acceptability, using qualitative content analysis [46], among the participants who completed structured exit interviews. We used an open-coding, group-based process to generate emergent themes on areas of strength and weakness in the app that would complement our quantitative usability and engagement data. The first author (DR) read through all responses and crafted the initial codebook. JA and DR then jointly coded the first 4 interviews (4/41, 10%) during research meetings to refine the codebook and achieve full consensus on code definitions and inclusion and exclusion criteria. Special attention was paid to create codes for deviant opinions in the responses. DR then coded the remaining interviews using a constant comparison technique to examine how newly coded texts matched previously coded information. Coding was completed manually.

JA reviewed the coding process iteratively to assure a consistent code app. DR and JA then grouped the responses and comments thematically over several research meetings. Acceptability feedback on the app’s 3 main features guided the process of generating themes, although we paid special attention to searching for unexpected ideas as well. JMS and LJ, who were familiar with the participants’ responses but did not participate in the coding process, then reviewed the derived themes to assure the validity and interpretation of the themes.

We analyzed engagement data captured directly from the app on all participants through the following assessments. We calculated the mean number of check-ins (out of 8 possible opportunities) for all the enrolled participants. We also dichotomized the check-in variable, based on participants’ median number of check-ins (7; IQR 5-8), with high use representing 7 or 8 check-ins and low use representing 0 to 6 total check-ins. We then determined the proportion of goal modifications and the types of goals selected. We also evaluated the frequencies of comments left and messages sent and how many participants sent a message in the study, commented during the study, chose to share their data with social ties or all users, and checked in after their 8-week study period was over.

In secondary analyses, we estimated associations between participant baseline characteristics and participant reported ease of use and app engagement, specifically high (7-8 check-ins) versus low (0-6 check-ins), in unadjusted logistic regression models and age- and sex-adjusted models. In addition, we examined preliminary results of app engagement by the following: (1) reported success in achieving goals; (2) coding and content analysis of comments within the app; (3) change in cancer prevention knowledge from baseline using two-tailed, paired *t* tests; and (4) change in self-reported behaviors from baseline using two-tailed, paired *t* tests. Coding and content analysis of comments were performed using the same processes as the open-ended feedback. All quantitative analyses were conducted using Stata version 15.1 (StataCorp LLC).

## Results

### Overview

**Figure 2:**
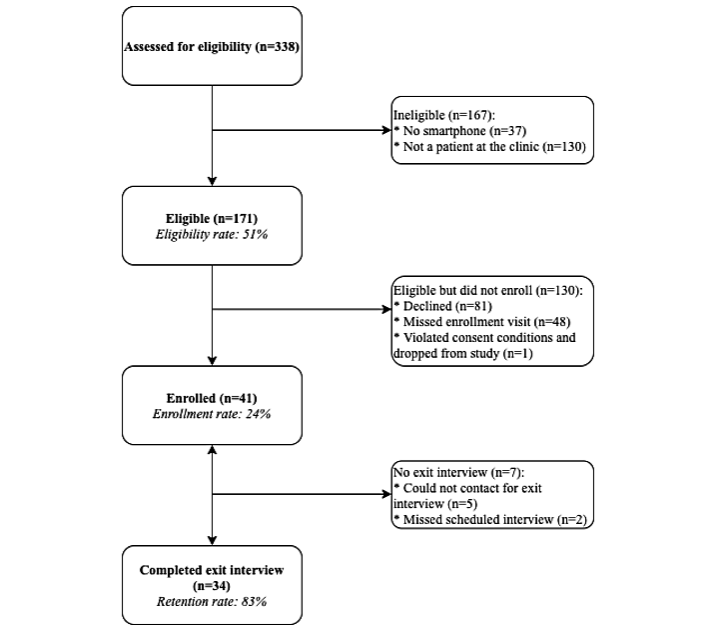
CONSORT (Consolidated Standards of Reporting Trials) diagram with enrollment and retention rates.

Of the 338 individuals who were approached in the clinic waiting rooms, 171 (50.6%) met the eligibility criteria. Of those eligible, 23.9% (41/171) completed the enrollment survey, downloaded the app, and consented to have us track their use of the app. Of the 41 enrolled participants, 34 (83%) completed the exit survey after using the app for 2 months (Figure 2).

The average age of the participants was 51 (SD 12) years, and 76% (31/41) were women. Of the 41 participants, 31 (76%) reported tracking their health before the study, with 18 (44%) using some form of technology to do so. Most participants (28/41, 68%) relied on friends and family to accomplish a health goal within the past year, and 68% (28/41) participants were comfortable sharing *some* or *a lot* of their personal health information with friends and family. Conversely, fewer participants (11/41, 27%) were comfortable discussing personal health topics on the web (Table 1).

**Table 1:** Participant baseline characteristics (N=41).

Characteristics				Values
**Demographics**				
	Age (years), mean (SD)			51 (12)
**Participants, n (%)**				
	Females			31 (76)
**Technology use, n (%)**				
	**Smartphone operating system**			
		Apple operating system	17 (41)	
		Android operating system	23 (56)	
		Missing	1 (2)	
	**Average use of phone**			
		Twice or more per day	40 (98)	
		Nearly daily or daily	1 (2)	
**Comfort sharing health information, n (%)**				
	Discusses health topics on the web			12 (29)
	Discusses personal health on the web			11 (27)
	**How much health information was shared with friends or family?**			
		None	4 (10)	
		A little	9 (22)	
		Some	6 (15)	
		A lot	22 (54)	
	**How many friends or family share health information?**			
		None	1 (2)	
		1-5	18 (44)	
		5-10	14 (34)	
		10-15	4 (10)	
		>15	4 (10)	
**Health behaviors, n (%)**				
	Set a health goal within last month			30 (73)
	Currently tracking health			31 (76)
	**Methods have used to track health**			
		Uses technology to track health (eg, phone app, eHealth tracker, and patient portal)	18 (44)	
		Tracks health manually (eg, health journal)	16 (39)	
	Relied on friends or family to accomplish the health goal in previous year			28 (68)

### Usability and Acceptability of Mobile App

**Figure 3:**
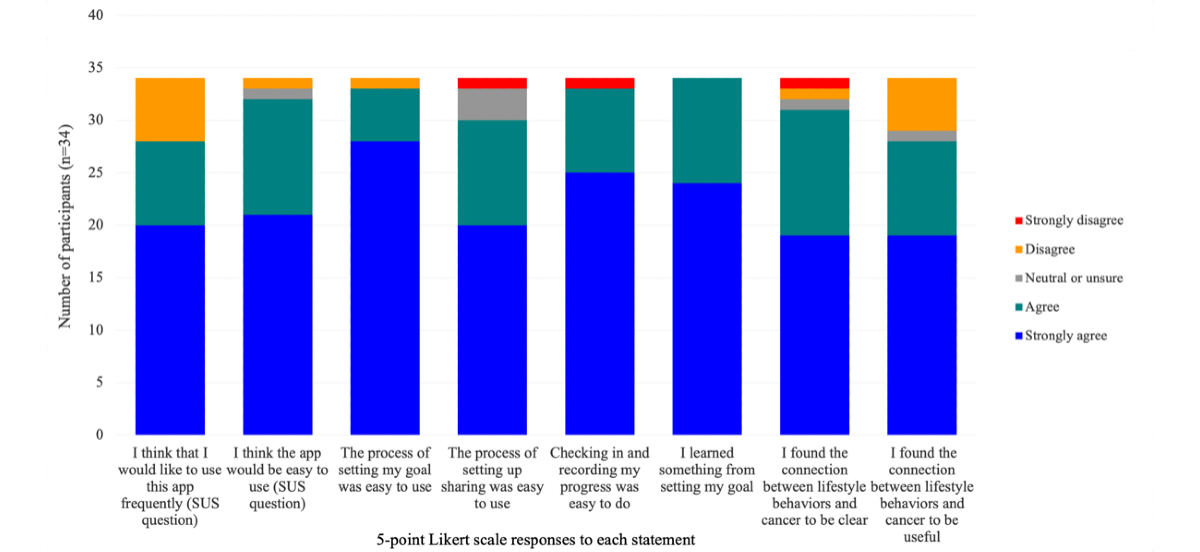
Participant feedback by feature. The first two questions are taken from the System Usability Scale.

The majority of participants who completed the exit survey (82%, 28/34) reported positive experiences with the app. The app’s mean SUS score was 87 (SD 12; median 90; IQR 78-95). Specifically, 94% (32/34) of the participants agreed that the app was easy to use and 82% (28/34) agreed that they would like to use the app frequently. Figure 3 illustrates the distribution of responses to the Likert scale statements about app feature ease of use and knowledge delivery.

Analysis of open-ended responses revealed several key themes depicting both excitement about the app and opportunities for app refinements, with many participants expressing the need for more personalized feedback and social interactions (Textbox 1).

**Themes from participant open-ended feedback on app acceptability.**

**Ease of use is a major strength**

- “It was very helpful. Simple. I don’t get a lot of notifications which was great.” [Participant 7]
- “Not difficult at all. It was easy to do it on the go. Very convenient.” [Participant 32]

**Features encourage app use and motivate behavioral change**

- “The text message reminders were helpful and it keeps people on track.” [Participant 31]
- “...it is encouraging to see other people doing what you’re doing. It felt like you were doing it as a group.” [Participant 15]
- “The goal options were good and [it] helped me change my lifestyle.” [Participant 24]

**Request for more avenues for social interaction**

- “I would like meetings for help setting up these goals [and] to share goals and conquests.” [Participant 2]
- “It would have been nice to be able to talk more about goals with others in the app.” [Participant 22]

**Request for personalized feedback to facilitate goal completion**

- “Give us recipes for healthier foods would be nice.” [Participant 3]
- “So when I didn’t meet my goal I wish that there were tips given to me or more info to help me achieve it next.” [Participant 22]

### Engagement

**Figure 4:**
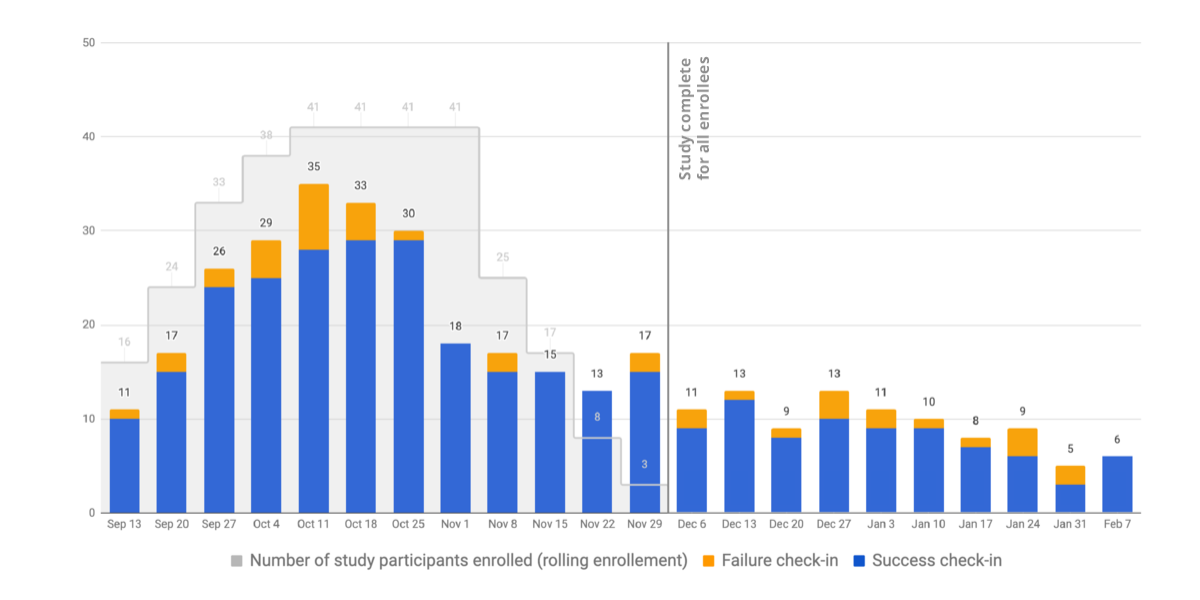
App engagement during and after the 8-week study period.

We captured participant engagement with the app using check-ins during and after the 8-week study period, as depicted in Figure 4. Out of 8 possible weekly check-ins during the study period, the mean number of check-ins per participant was 5.7 (SD 2.7). Of the total number of participants, 76% (31/41) participants checked in at least four times, 56% (23/41) checked in seven or eight times, and 10% (4/41) never checked in after downloading the app. Participants continued to receive check-in invitations after they completed the 8-week study period, and 51% (21/41) participants checked in at least once during the postintervention period.

**Figure 5:**
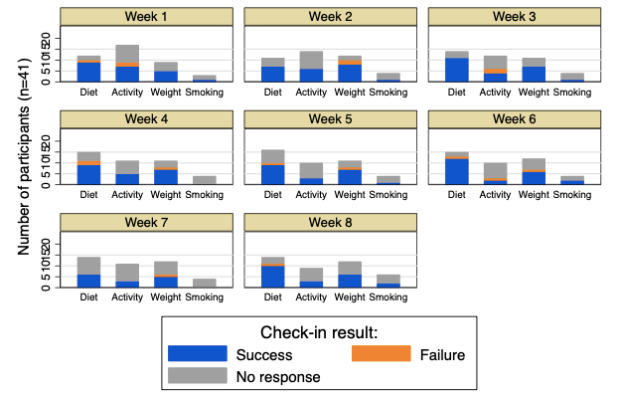
Goal selection and attainment by goal type and study week.

In terms of goal selection, activity-related goals were initially the most commonly selected, but participants switched to diet-based and weight-tracking goals as the study progressed. Regular users (31/41, 76%) on average set 2.1 goals during the 8 weeks. Participants who selected diet or weight-tracking goals were also more likely to check in than participants who selected activity or smoking cessation goals (Figure 5).

Among the 41 participants, 34 (83%) opted to share progress with their social ties and 18 (44%) opted to share their progress with all app users. A majority of participants used the app’s comments box (29/41, 71%), which allowed participants at each check-in to reflect on their weekly progress. Fewer participants (8/41, 20%) used the app’s messaging feature, possibly because of the incomplete functionality of this feature in the beta version of the app. Participants left a total of 111 comments and sent 26 messages during the study period. In these comments, participants described both facilitators and barriers to goal success, left inspiring messages, and linked goal success to overall improvements in their health and well-being (Textbox 2). Of those 34 participants who completed the exit interview, 23 (68%) reported visiting the app at least once outside the weekly check-in to view other users’ progress or read the health information on the app.

**Themes on how participants used the comment section of the app.**

**Identified barriers that led to an unsuccessful week**

- “Hi, I’ve just been super busy, sometimes not stopping to eat until the evening. Thanks for contacting me. I needed a reminder.” [Participant 32]
- “I got weak and started craving french fries. They just were so tempting and I failed but I know I’ll be able to get past these cravings.” [Participant 40]

**Described facilitators of weekly goal success**

- “I had to push myself, even when I didn’t want to do it. I am not a excise person that [goes] to the gym so I just start walking around my block 2 times. Then when it got easy then I add more times that I walk around my block.” [Participant 34]
- “I have been determined to keep to my goal of lessening processed foods. I hope to continue this as well as engaging my family. Thank you.” [Participant 22]
- “I was successful because I had help from my friends to work out.” [Participant 42]

**Left inspiring messages**

- “I try to keep my eyes on the gold.” [Participant 14]
- “Everything is good if you stay positive will be good.” [Participant 2]

**Detailed impact on health and overall well-being**

- “Me and my grandkids play in the park. I even try to run a little bit. Still no smoking. Feel good.” [Participant 2]
- “Accountability, My Fitness Pal, drop in lbs. & new burst of energy! Liking this New Me!” [Participant 4]
- “I actually increased my walk to 45 min. The more I do the more motivated I become. I have also started doing 20 min of stretches for seniors. Youtube...first thing after tea in morning before walk. I’m loving it!” [Participant 25]

**Celebrated success and major health improvements**

- “I have lost 45 pounds and I feel great.” [Participant 2]
- “I really had to really work on me to stop smoking but I did it.” [Participant 28]

### Factors Associated With App Engagement

We did not find any participant baseline characteristics to be significantly associated with app engagement. We found significant univariate associations between low app engagement and loss to follow-up, as well as low app engagement and the belief that the app was too complicated, although these associations disappeared after age and sex adjustment (Table 2).

**Table 2:** Differences in app engagement by participants’ characteristics (N=41).

Characteristics				Low app user (0-6 total check-ins; n=18), n (%)		High app user (7-8 total check-ins; n=23), n (%)		Unadjusted OR^a^ (95% CI) for high app engagement		Adjusted OR (95% CI) for high app engagement^b^	
**Baseline characteristics**											
	**Sex**										N/A^c^
		Female	16 (89)		15 (65)		Reference				
		Male	2 (11)		8 (35)		4.27 (0.78-23.4)				
	**Age (years)**										N/A
		20-39	5 (28)		2 (9)		Reference				
		40-59	10 (56)		13 (57)		3.25 (0.52-20.37)				
		60-79	3 (17)		8 (35)		6.67 (0.81-54.96)				
	**Health tracking**										
		Does not track health	3 (17)		4 (17)		Reference		Reference		
		Tracks health manually	7 (39)		9 (39)		0.96 (0.16-5.80)		1.21 (0.15-9.48)		
		Tracks health with technology	8 (44)		10 (43)		0.94 (0.16-5.46)		2.89 (0.31-26.71)		
**Postuse characteristics**											
	**Filled out an exit survey**										
		No	6 (33)		1 (4)		Reference		Reference		
		Yes	12 (67)		22 (96)		11 (1.18-102.4)^d^		6.11 (0.59-63.3)		
	**Thought app was too complex (n=34)**										
		No	8 (75)		21 (95)		Reference		Reference		
		Yes	4 (25)		1 (5)		0.10 (0.01-0.99)^d^		0.10 (0.01-1.15)		
	**Thought app was too simplistic (n=34)**										
		No	11 (92)		18 (82)		Reference		Reference		
		Yes	1 (8)		4 (18)		2.44 (0.24-24.8)		1.49 (0.12-17.7)		

^a^OR: odds ratio.

^b^Adjusted for sex and age (as a continuous variable).

^c^N/A: not applicable.

^d^Statistically significant result at *P*<.05.

### Implications of App Engagement on Goal Success and Behavioral Change

Most participants reported accomplishing their selected health goals each week, with the average participant succeeding in 5.1 out of the 8 weeks (SD 2.7). Of the 328 total check-in opportunities (41 participants×8 weeks), 211 (64.3%) were marked as successful. Participants selected a failure only 6.7% (22/328) of the time, with the other 28.9% (95/328) of opportunities yielding no response. Using two-tailed, paired *t* tests, we found that cancer prevention knowledge increased from baseline, with participants being more likely to recognize the link between the lack of exercise and unhealthy weight with cancer. Two-tailed, paired *t* tests also showed an improvement in diet scores among participants who selected a diet-related goal. We also found that 25% (2/8) of the participants who selected smoking cessation as their goal no longer reported smoking at the exit interview. As noted in Textbox 2, the open-ended comments that participants left after each check-in also suggested that the app was able to motivate behavioral change.

## Discussion

### Principal Findings

Prior meta-analyses reveal mixed evidence about the ability of health promotion mobile apps to change behavior in a quantifiable manner (eg, significantly increase physical activity) [47] or improve tangible health outcomes (eg, reduce blood pressure or cholesterol levels) [48]. This may be in part because of the fact that many apps are developed without user testing or constructed without a coherent behavior change framework [11,12]. In this pilot study, we examine the Healthier Together mobile app, which was developed through iterative user testing and is grounded in social network and goal-setting theories of behavior change. We found that the app strongly engaged our target population, even beyond the study period, with promising results on participants’ knowledge of cancer prevention behaviors and success in achieving their cancer prevention behavioral goals.

Consistent with prior research showing that minority populations frequently use their mobile phones to obtain health information [15,16], almost half of the participants in our study (18/41, 44%) used various forms of technology to track their health before participation. Participants in the study also reported that they were comfortable discussing their health with their social networks. After interacting with the Healthier Together app for 2 months, the participants supported the usability and acceptability of the app. The app’s mean SUS score of 87 indicates that the app is very usable [42], and the vast majority of participants (28/34, 82%) indicated that they would like to use the app frequently if available. The open-ended responses further showcased the participants’ beliefs in app acceptability and overall utility. For example, the fact that nearly all participants identified one or more of the app’s features as facilitators of goal success shows that the participants understood and used the theoretical basis of the app to their advantage.

Our findings suggest that the high usability scores and acceptability of this app by participants translated into their high engagement: most participants missed only 1 check-in out of 8 and over 50% (21/41) continued to check in after the 8-week study was over. This high engagement is especially encouraging, as participants were not incentivized to engage with the app and only incentivized to complete the baseline and exit surveys. Engagement with the app extended beyond weekly check-ins to report goal achievement. Most participants (23/34, 64%) logged on to the app outside of the weekly check-in to explore the app’s other features, whereas many users (29/41, 71%) left unprompted comments when they checked in to highlight their progress or troubleshoot barriers to goal success.

Our study suggests that app engagement, in turn, appears to motivate behavioral change. First, most participants reported accomplishing their weekly health goals. On average, participants reported success in goal attainment of 64.3% (211/328) of the time. These successes represent health behaviors that the participants may not have undertaken if not enrolled in the study. Through the comments participants left when checking in, we found evidence of how these successes translated to tangible behavior changes and health outcomes: participants reported building up exercise programs, eating healthier foods, losing a significant amount of weight, and smoking cessation. We were able to partially capture these effects quantitatively, with participant diet scores and cancer prevention knowledge increasing after the study. Future work should enlist a larger sample size and conduct a randomized controlled trial to further evaluate the effectiveness of this intervention as well as determine what participant characteristics may better forecast app engagement. In this study, we found that neither age nor prior health tracking methods predicted app engagement, which may suggest that the app has a broad appeal.

This study also yielded important data to improve the Healthier Together app. For example, many participants wanted the app to both provide them with additional feedback about how to attain their health goals and facilitate easier ways to connect with other users working on similar goals. We hope that the future iteration of the Healthier Together app will have a group chat feature that allows users to work on goals collectively with other users, potentially with the input of a health provider that occasionally checks the chat. Research suggests that decentralized social networks, such as those envisioned by Healthier Together, can harness social influence to amplify social learning and enhance group intelligence on topics including finance and health [27]. Therefore, rather than the Healthier Together staff providing individualized feedback to each user, users could solicit feedback and recommendations from their peers.

Notably, of the 328 check-ins, there were 22 reported failures, 211 successes, and 95 nonresponses, suggesting that participants were more likely to check in if they met their weekly goal. This may be explained by a number of cognitive biases, such as social desirability bias, with participants not wanting to publicly admit an unsuccessful week [49]. Future versions of the app should aim to reframe unsuccessful weeks as opportunities for feedback and goal reflection rather than as reasons to disengage from the app. This study also found a lower rate of engagement with the app when users selected activity-based goals and an overall shift from activity-based goals to diet-based and weight-tracking goals as the study progressed. One potential reason for this observation is that the study occurred during the fall of 2019 in Philadelphia, and the colder weather may have disincentivized some participants from exercising [50]. Participants may have also been more conscious of their diet, given the upcoming holiday season [51]. Future versions of the app will aim to adjust the predetermined SMART goals to account for such variations.

### Strengths and Limitations

The success of this study in showing Healthier Together is engaging, usable, and acceptable and in providing the development team with important feedback for future versions of the app must be framed within certain limitations. First, this study was not powered to detect changes in health behavior among participants, nor did it randomize participants to test its effectiveness. The focus of this pilot study on usability and acceptability rather than effectiveness is commonplace for the initial stages of app development [52,53] and is consistent with the use of an iterative process to build health apps [35]. Quantitative analysis of close-ended questions in this study was descriptive and designed to inform future inquiries. Second, we focused on recruiting non-Hispanic Black primary care patients using purposive sampling and could not generalize these results if Healthier Together was downloaded in a nonclinical setting. However, we chose this methodology given the objective of Healthier Together is to facilitate cooperation between primary care patients and their providers on health goals. We did not independently code our structured interviews and, therefore, did not generate an intercoder reliability statistic [54]. Our analytic approach allowed us to better generate themes that enhanced our primary quantitative usability and engagement data. We also took the following key steps to assure the validity of the coding process consistent with external guidelines: (1) 2 coders achieved full consensus on code definitions reaching consensus on any deviant opinions, and (2) 2 additional independent researchers reviewed and edited the themes [55].

### Conclusions

In conclusion, this study showed that non-Hispanic Black primary care patients found Healthier Together, an evidence-based mobile app that focuses on cancer prevention behaviors, both engaging and valuable. We hope that the results of this study will inform future research on the development of health behavior interventions for minority populations, especially those that aim to leverage goal setting, social cooperation, and health technology.

## Abbreviations

PCP: primary care provider
REDCap: Research Electronic Data Capture
SMART: specific, measurable, achievable, realistic, and time-bound
SUS: System Usability Scale
